# Cancer patient management strategy in a Cancer Center of Zhejiang, China during the COVID-19 pandemic

**DOI:** 10.1186/s12885-020-07577-8

**Published:** 2020-12-07

**Authors:** Songxiao Xu, Xiangdong Cheng, Zhiwen Pan, Qian Song, Yihong Wang, Juan Xiong, Yongyi Chen, Fan Fan, Jing Zhu, Wanying Wu, Xueying Deng, Yanpin Yu, Xiaohong Xu, Wenhu Chen, Tao Zhu, Yang Yu, Kaizhong Liu, Guoliang Shao, Ming Chen, Enyan Yu

**Affiliations:** 1 Department of Clinical Laboratory, Cancer Hospital of the University of Chinese Academy of Sciences (Zhejiang Cancer Hospital), Hangzhou, China; 2 Institute of Cancer and Basic Medicine (IBMC), Chinese Academy of Sciences, Hangzhou, China; 3 Department of Gastric Surgery, Cancer Hospital of the University of Chinese Academy of Sciences (Zhejiang Cancer Hospital), Hangzhou, China; 4 Department of Pathology, Sir Run Run Shaw Hospital, School of Medicine, Zhejiang University, Hangzhou, China; 5 Department of Nursing, Cancer Hospital of the University of Chinese Academy of Sciences (Zhejiang Cancer Hospital), Hangzhou, China; 6 Department of Radiology, Cancer Hospital of the University of Chinese Academy of Sciences (Zhejiang Cancer Hospital), Hangzhou, China; 7 Department of Gynecologic Oncology, Cancer Hospital of the University of Chinese Academy of Sciences (Zhejiang Cancer Hospital), Hangzhou, China; 8 Department of Breast Surgery, Cancer Hospital of the University of Chinese Academy of Sciences (Zhejiang Cancer Hospital), Hangzhou, China; 9 Department of Critical Care Medicine, Cancer Hospital of the University of Chinese Academy of Sciences (Zhejiang Cancer Hospital), Hangzhou, China; 10 Department of Radiation Oncology, Cancer Hospital of the University of Chinese Academy of Sciences (Zhejiang Cancer Hospital), Hangzhou, China; 11 Department of Clinical Psychology, Cancer Hospital of the University of Chinese Academy of Sciences (Zhejiang Cancer Hospital), Hangzhou, China

**Keywords:** Cancer, COVID-19, Infection-control interventions

## Abstract

**Background:**

Due to the increased risk of viral infection and the severe shortage of medical resources during the pandemic of COVID-19, most hospitals in the epidemic areas significantly reduced non-emergency admissions and services, if not closed. As a result, it has been difficult to treat cancer patients on time, which adversely affects their prognosis. To address this problem, cancer centers must develop a strategic plan to manage both inpatients and outpatients during the pandemic, provide them with the necessary treatment, and at the same time prevent the spread of the virus among patients, visitors and medical staff.

**Methods:**

Based upon the epidemic situation in Zhejiang Province, China, the number of running non-emergency medical wards in the Zhejiang Cancer Hospital was gradually increased in a controlled manner. All staff of the hospital received COVID-19 preventive training and was provided with three different levels of protection according to the risks of their services. Only patients without a known history of SARS-CoV-2 contact were eligible to schedule an appointment. Body temperature was measured on all patients upon their arrival at the hospital. Chest CT image, blood cell counting and travel/contact history were investigated in patients with fever. Respiratory tract samples, such as sputum and throat swabs, from all patients, including those clinically suspected of SARS-CoV-2 infection, were collected for nucleic acid detection of SARS-CoV-2 before treatment.

**Results:**

A total of 3697 inpatients and 416 outpatients seeking cancer treatment were enrolled from February 1 to April 3, 2020, in compliance with the hospital’s infection-control interventions. The clinicopathological parameters of the patients were summarized herein. 4237 samples from 4101 patients produced negative RNA testing results. Four clinically suspected patients all presented negative RNA test results and were excluded from the SARS-CoV-2 infection through follow-up retesting and monitoring. Seven patients with only N-gene positive results were retested, followed by CT scan and SARS-CoV-2 contact history investigation. All of them were finally diagnosed as non-infected patients. There was one outpatient who was confirmed positive by virus RNA test and then followed up. She might be an asymptomatic laboratory-confirmed case. During the study period, there was no SARS-CoV-2 infection among staff, patients and escorts of patients in the Zhejiang Cancer Hospital.

**Conclusion:**

This study suggested our infection-control interventions, including viral nucleic acid test, could be used as a reliable method to screen cancer patients in the area with moderate COVID-19 prevalence. Cancer may not be a high-risk factor of SARS-CoV-2 infection.

## Background

A novel severe acute respiratory syndrome coronavirus 2 (SARS-CoV-2), causing coronavirus disease 2019 (COVID-19) emerged and rapidly spread throughout the whole world [1–4]. As of April 172,020, a total of 2,100,272 COVID-19 cases have been confirmed worldwide, and the disease has become a critical global public health issue (https://www.who.int/docs/default-source/coronaviruse/situation-reports/-sitrep-74-covid-19-mp.pdf?sfvrsn=4e043d03_4). Furthermore, unconfirmed infected patients exist due to the asymptomatic (subclinical) infection and the incubation period [5, 6].

The secondary symptoms of COVID-19 patients are headache, diarrhea, nausea, and vomiting, which highly resemble the symptoms of cancer patients receiving chemotherapy and/or immunotherapy [2, 7–9]. Moreover, cancer patients are particularly susceptible to pneumonia due to a weakened immune response to pathogens, such as bacteria and virus [10–12]. Therefore, during the epidemic of COVID-19, the oncologists faced a significant challenge to distinguish infected from non-infected patients [13, 14]. These difficulties may cause cancer hospitals to reduce non-emergency medical wards and oncology service to decrease the risk of virus transmission, despite that it can delay the scheduled chemotherapy or surgery for cancer patients. Delayed treatment will worsen the patients’ prognosis and may sometimes lead to severe consequences. A viewpoint from the Chinese National Cancer Center recommended that several mandatory measures were carried out during the COVID-19 pandemic. However, the cancer patients received treatment in their hospital were mainly outpatients (2795 outpatients vs. 149 inpatients) and very few patients were tested by SARS-CoV-2 nucleic acid analysis [15].

Taking into account the potential of COVID-19 of nosocomial transmission to cancer patients and medical staff and the ability of SARS-CoV-2 causing fatal pneumonia, our hospital called on all medical professionals to learn the medical knowledge of COVID-19 and developed a set of infection-control interventions. After that, the hospital strictly followed the intervention protocols with an adequate screening of the nucleic acid of SARS-CoV-2 from February 1 to April 3, 2020.

Additionally, whether cancer serves as an independent risk factor for COVID-19 infection remains unclear. The prevalence of COVID-19 infection was recently reported being higher in a cancer patient cohort than in individuals without cancer [16]. However, among the 18 infected cancer patients in the cohort, 12 patients showed no signs of a weakened immune response, which did not well represent the immune status of the majority of patients in recovery from cancer therapy. Also, there were 2 cancer patients with unknown cancer treatment information [17, 18]. We could suspect that the infection of COVID-19 in the 12 cancer survivors was not related to cancers. Additionally, the infected cancer patients were older than other patients in this cohort of 1590 cases, which indicated that age might be an essential factor for their vulnerability and hospitalization due to more severe symptoms as compared to younger patients. A letter in *The JAMA Oncology* suggested that cancer patients at Zhongnan Hospital of Wuhan University harbored a higher risk of COVID-19 than the community in Wuhan. However, hospital-acquired transmission cannot be excluded in these patients in Wuhan [19]. Despite that, the results of COVID-19 screening for cancer patients outside Wuhan are still unknown. Because of the different prevalence of COVID-19 between Wuhan and most other regions of China and other countries, it is necessary to answer the question of whether cancer patients in moderate epidemic areas carry a higher risk of COVID-19 infection than the general population in the community.

Thus, to prevent and control COVID-19 infection among cancer patients, and to better manage cancer patients during the pandemic, we present here our management of infection-control interventions and SARS-CoV-2 RNA screening results with the clinical features of suspected COVID-19 cases at Zhejiang Cancer Hospital, Hangzhou, China.

## Methods

### Staff training and 3-level protection

All personnel, including medical professionals and supporting staffs, received comprehensive training on COVID-19 pandemic prevention and control. Three different levels of protection were provided to our staff. The detail of protective measures and allowed practices were showed in Supplementary Table 1.

### Patient enrollment

**Fig. 1:**
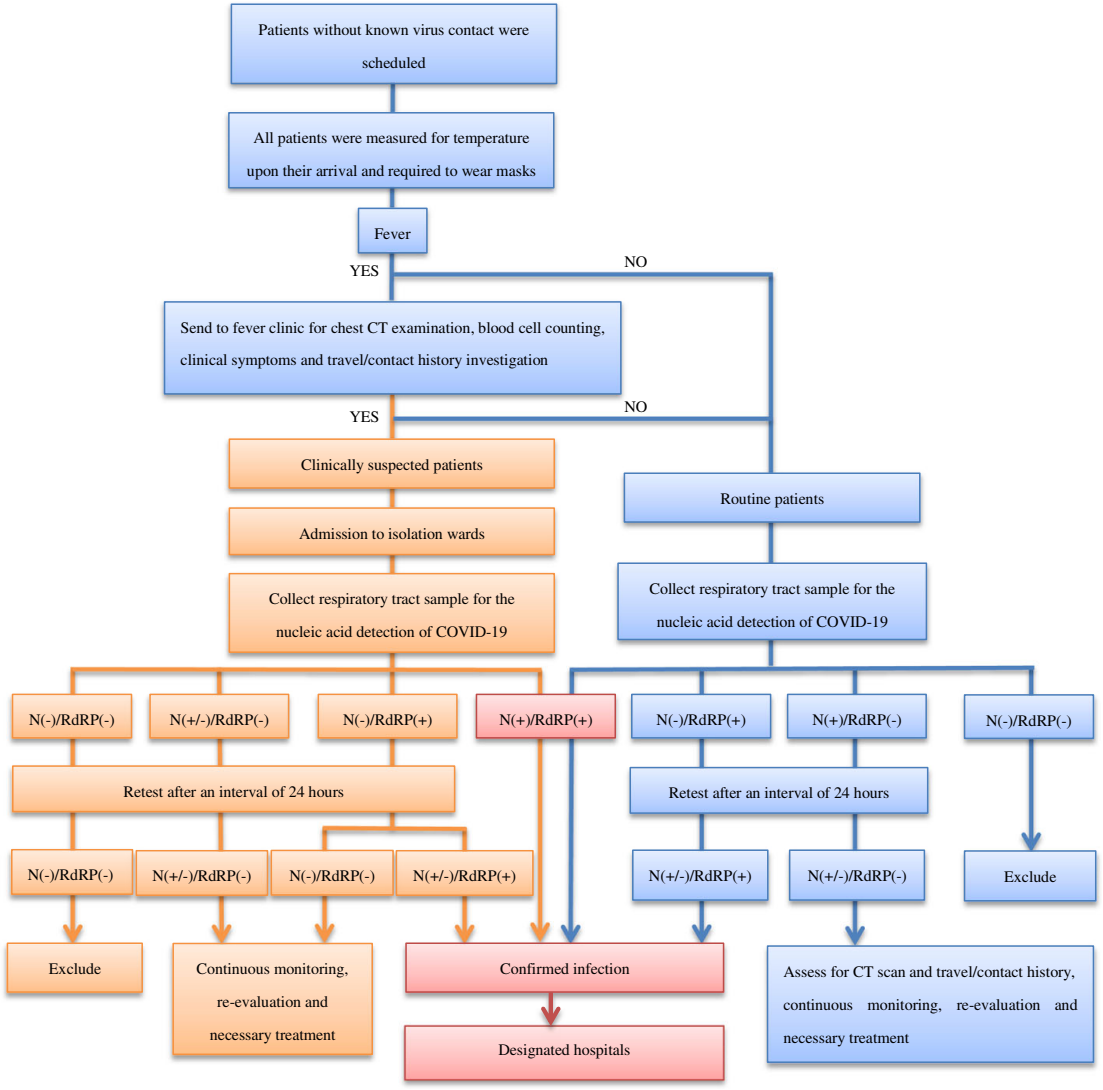
Infection-control interventions for hospital admissions during the outbreak of COVID-19

During the epidemic of COVID-19 from February 1 to April 3, 2020, all patients need to schedule an appointment to see the doctor through the online hospital booking system or by phone. SARS-CoV-2 contact history was investigated through the booking system. Only patients without a known history of SARS-CoV-2 contact were eligible to schedule an appointment. The patients’ body temperatures were measured upon their arrival at Zhejiang Cancer Hospital during the epidemic of COVID-19 from February 1 to April 3, 2020. All the patients were advised to wear masks during the visitation in our hospital. Individuals with fever were sent to fever clinic for travel history investigation, chest CT examination, blood cell counting and clinical symptoms assessment, such as cough, myalgia, fatigue. Based on the above information, suspected patients could be clinically diagnosed according to the updated COVID-19 Diagnosis and Therapy Guideline from Center of Disease Control (CDC), China, 7th Edition (http://www.chinacdc.cn/jkzt/crb/zl/szkb_11803/jszl_11815/202003/W020200305456621460977.pdf). Before further surgery, radiation, or chemotherapy, respiratory tract samples (sputum and/or pharyngeal swab) from all patients, including clinically suspected ones, were collected for the SARS-CoV-2 RNA testing. In the case of suspected patients, oncologists wear level-3 protections to perform routine therapeutic activities including chemotherapy, radiation, and surgery in our hospital. When those patients were diagnosed with infection, they were sent to a designated hospital in the area for anti-infection treatment and follow-up cancer related treatment. The infection-control interventions for hospital admissions during the outbreak of COVID-19 were presented in Fig. 1.

### RNA test and following up evaluation

A real-time PCR assay (Liferiver, Z-RR-0479-02-50, China) was used to analyze all the patient samples, which was the first National Medical Products Administration (NMPA) approved SARS-CoV-2 RNA testing method in China. The protocol of this real-time RT-PCR assays targeting the RNA-dependent RNA polymerase (RdRp), nucleocapsid (N) and envelope (E) genes of SARS-CoV-2. Only RdRp and N genes were considered according to COVID-19 Prevention and Control Guideline from CDC, China, 5th Edition (http://www.chinacdc.cn/jkzt/crb/zl/szkb_11803/jszl_11815/202002/W020200223341563346503.pdf). Ct value 43 is the cutoff value to differentiate positive and negative amplifications. Patients with both positive RdRp and positive N genes were confirmed as infected patients. Clinically suspected patients need to be retested after 24 h if the first round of test results were negative or equivocal. If a patient had only one gene-positive among these two genes, this patient needed to be retested after 24 h interval. CT scan and travel or contact history will be thoroughly investigated. If the RdRp gene was tested positive again, the patient was infected by COVID-19. If the N gene was tested positive twice, the patient became a laboratory suspected one due to the specificity of N gene to COVID-19 was relatively low [20]. CT scan, travel or contact history investigation and follow up tests are required to clarify a laboratory suspected patient.

### Statistical analysis

Continuous data were directly expressed as a range. Categorical data were expressed as number and percentage. The SPSS software (version 19.0) was utilized for statistical analysis.

### Role of the funding source

The study sponsors had no role in study design, data analysis, data interpretation, writing of the report, or the decision to submit the paper for publication. The corresponding author had full access to all data in the study and had final responsibility for the decision to submit for publication.

## Results

According to the most updated information by April 172,020, none of the staff in Zhejiang Cancer Hospital or visitors was infected by SARS-CoV-2 during the period of this study.

**Fig. 2:**
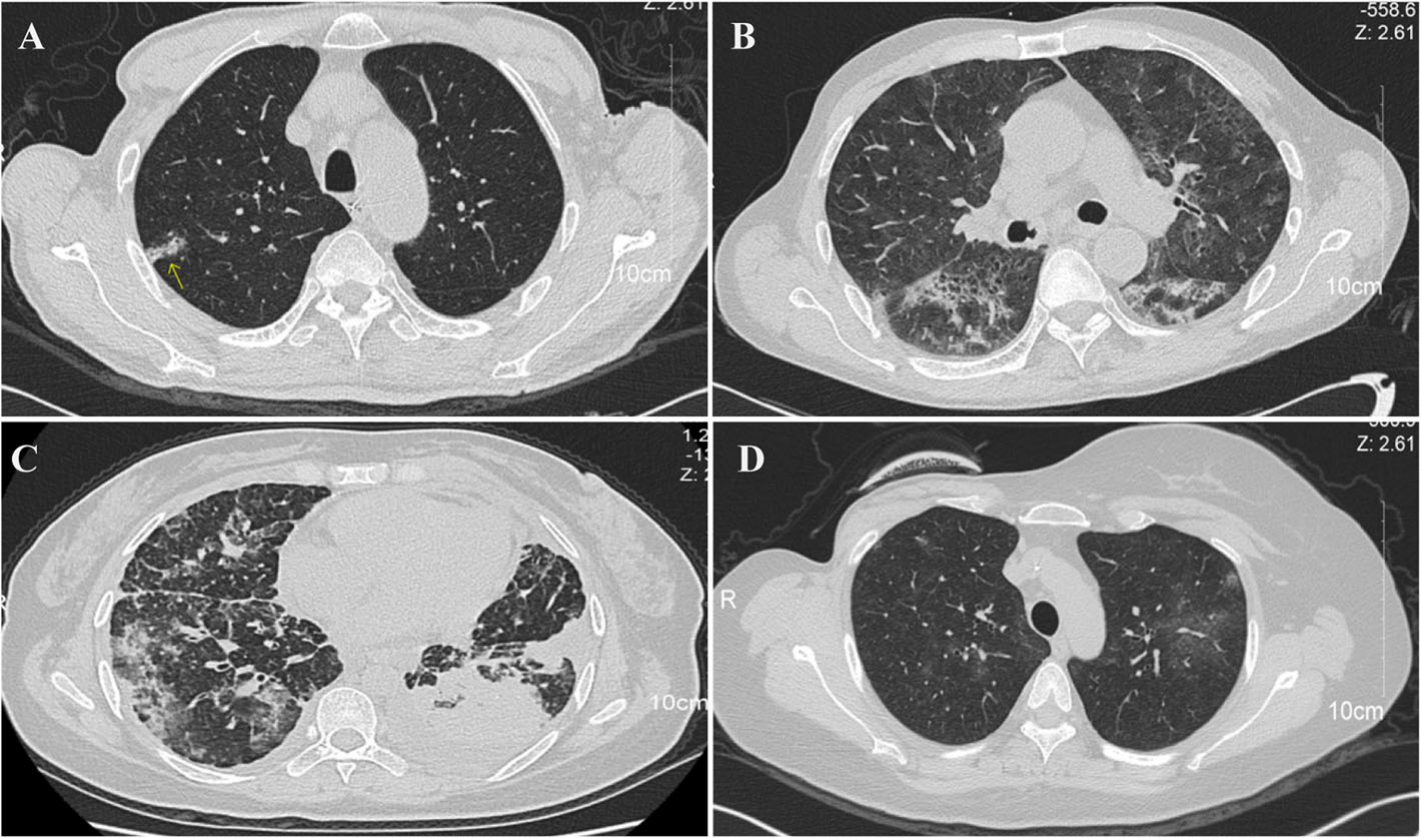
Chest CT images (transverse plane) of 4 clinical suspected COVID-19 patients. **a** Patient 1: right-sided multiple ground-glass opacities. **b** Patient 2: bilateral multiple ground-glass opacities. **c** Patient 3: bilateral multiple ground-glass opacities. **d** Patient 4: bilateral ground-glass opacities. Ground-glass opacities were indicated by an arrow in (A)

A total of 4113 patients were enrolled from February 1 to April 3, 2020. Their clinicopathologic information was summarized in Table 1. The residence of all the patients was summarized in Table 2. The number of local confirmed infected cases were obtained from the biggest real-time reporting system of nCOV in China, which has been visited by people over 3.6 billion times since the start of this epidemic (http://ncov.dxy.cn/ncovh5/view/pneumonia). Four thousand two hundred thirty-seven samples from 4101 patients produced negative RNA testing results. There were 4 clinically suspected COVID-19 cases before RNA testing, including 1 esophageal cancer patient, 1 hypopharyngeal cancer patient, 1 lung cancer patient, and 1 breast cancer patient. All these 4 patients presented with a fever. Three patients showed other upper respiratory symptoms. Laboratory examination suggested that none of the 4 suspected COVID-19 cases had a low leukocyte count (< 9.5 ×  10^9^ cells per L). Three patients had lymphopenia (< 1.0 × 10^9^ cells per L). Three patients had an increased level of C-reactive protein (CRP) (> 10 mg/L). Two patients had an elevated concentrations of lactate dehydrogenase concentrations (LDH) (> 240 U/L). One patient had an elevated level of alanine aminotransferase (ALT) (> 50 U/L) and an increased concentration of aspartate aminotransferase (AST) (> 40 U/L). All these 4 patients showed signs of viral infection on CT images (Fig. 2a-d). Their detailed information was present in Table 3. Patient1 was N-gene positive after the first round of PCR testing. After 24-h interval, these patients were retested. All these patients except for patient1 were diagnosed as non-infected patients. Patient1 was excluded after 2 weeks of monitoring.

**Table 1:** Clinical features of all patients admitted to Zhejiang cancer hospital

Category	Total (***N*** = 4113)
Gender	
Male	1951(47.4%)
Female	2162(52.6%)
Age	
< 18 years	11(0.3%)
≥ 18 years and < 60 years	2283(55.5%)
≥ 60 years	1819(44.2%)
Patient types	
Inpatients	3697(89.9%)
Outpatients	416(10.1%)
Sample types	
Pharyngeal swab	1925(46.8%)
Sputum	2188(53.2%)
Cancer types	
Nasopharyngeal carcinoma	219(5.3%)
Head and neck cancer	205(5.0%)
Thyroid cancer	287(7.0%)
Esophageal cancer	207(5.0%)
Lung cancer	741(18.0%)
Gastric cancer	203(4.9%)
Liver cancer	125(3.0%)
Colorectal cancer	400(9.7%)
Cholangiocarcinoma	14(0.3%)
Abdominal tumor	20(0.5%)
Cervix tumor	80(1.9%)
Cervical cancer	335(8.1%)
Ovarian cancer	134(3.3%)
Breast cancer	385(9.4%)
Pelvic neoplasm	35(0.9%)
Connective tissue and soft tissue malignancies	65(1.6%)
Lymphoma	82(2.0%)
Brain cancer	42(1.0%)
Bladder cancer	34(0.8%)
Skin cancer	36(0.9%)
Prostate cancer	32(0.8%)
Kidney cancer	32(0.8%)
Vulvar cancer	23(0.6%)
Thymic tumor	20(0.5%)
Pancreatic cancer	61(1.5%)
Other cancer	97(2.4%)
Benign disease	40(1.0%)
Unknown	159(3.9%)

**Table 2:** Residence of all patients admitted to Zhejiang cancer hospital

City Names	Number of cancer patients	Confirmed COVID-19 cases in that city	Total population (million, 2018)
Wenzhou	211	504	8.287
Hangzhou	1225	181	7.741
Ningbo	197	157	6.030
Taizhou	323	146	6.054
Jinhua	319	55	4.890
Jiaxing	220	46	3.604
Shaoxing	407	42	4.472
Lishui	148	17	2.702
Quzhou	122	14	2.579
Huzhou	248	10	2.671
Zhoushan	18	10	0.969
Other Provinces	472		
Unknown	203

**Table 3:** Clinical and laboratory characteristics of suspected COVID-19

Clinical characteristics	Patient 1	Patient 2	Patient 3	Patient 4
Residence	Hangzhou	Hangzhou	Lishui	Shaoxing
Date of admission	1-Feb 2020	27-Feb 2020	9-Mar 2020	17-Mar 2020
Age (years)	78	56	42	43
Gender	Male	Male	Female	Female
Cancer type	Esophageal cancer	Hypopharyngeal cancer	Lung cancer	Breast cancer
Smoking status	Yes	Yes	No	No
Epidemiological history	Yes (Exposure to relevant environment)	No	No	No
Other diseases	No	No	Yes (Ovarian cancer and liver cancer)	No
Treatment history	Chemoradiotherapy 2 months ago	Chemoradiotherapy 2 months ago	Chemotherapy 1 month ago	Chemotherapy 3 weeks ago
Symptoms				
Fever	Yes(37.3 °C)	Yes(38.3 °C)	Yes(37.7 °C)	Yes(38.2 °C)
Cough	Yes	Yes	Yes	No
Fatigue or myalgia	No	Yes	Yes	No
Expectoration	Yes	Yes	Yes	No
Dyspnea	No	No	No	No
Headache	No	Yes	No	No
Diarrhea	No	No	No	No
**Laboratory characteristics**				
White blood cell count (× 10^9^ cells per L)	5	7.6	12.8	5.7
Low leukocyte count (< 9·5 × 10^9^ cells per L)	No	No	No	No
Lymphocyte count (× 10^9^ cells per L)	0.8	0.4	1.2	0.7
Lymphopenia (< 10^9^ cells per L)	Yes	Yes	No	Yes
CRP (mg/L)	11.12	31.04	12.25	9.21
Elevated CRP (> 10 mg/L)	Yes	Yes	Yes	No
LDH (U/L)	141	337	199	296
Elevated LDH (> 240 U/L)	No	Yes	No	Yes
ALT (U/L)	23	17	10	51
Elevated ALT (> 50 U/L)	No	No	No	Yes
AST (U/L)	26	30	25	48
Elevated AST (> 40 U/L)	No	No	No	Yes
**CT evidence of pneumonia**				
Typical signs of viral infection	Positive	Positive	Positive	Positive
1st round of PCR				
RNA-dependent RNA polymerase (RdRp)	Negative	Negative	Negative	Negative
Nucleocapsid (N) gene	Positive	Negative	Negative	Negative
2nd round of PCR after 24 h				
RNA-dependent RNA polymerase (RdRp)	Negative	Negative	Negative	Negative
Nucleocapsid (N) gene	Negative	Negative	Negative	Negative
**Follow-up Treatment**				
	Anti-infection therapy	Hormone therapy	Anti-infection therapy and Chemotherapy	Transfer to local hospital

*CRP* C-reactive protein. *LDH* Lactate dehydrogenase. *ALT* Alanine transaminase. *AST* Aspartate transaminase

Other 7 cancer patients were N-gene positive in the first round of test. Five of them were confirmed N-gene positive after retesting. Three patients showed similar clinical symptoms with COVID-19, such as cough, fatigue and expectoration. All 7 patients presented normal white blood cell count and C-reactive protein (CRP). One patient had lymphopenia (< 1.0 × 10^9^ cells per L). Four of them had abnormal LDH, AST or ALT levels. Patient 5 had positive CT findings (Table 4). This patient can neither be excluded from the viral infection nor be confirmed at that time due to the relatively low specificity of N-gene. She was transferred to a COVID-19 designated hospital in Hangzhou for follow-up monitoring. Fortunately, patient 5 with the other 6 patients were finally excluded from COVID-19 after at least 14 days of follow-up monitoring.

**Table 4:** Clinical and laboratory characteristics of positive N gene of SARS-CoV-2

Clinical characteristics	Patient 5	Patient 6	Patient 7	Patient 8	Patient 9	Patient 10	Patient 11
Residence	Hangzhou	Huzhou	Hangzhou	Heilongjiang	Hangzhou	Ningbo	Hangzhou
Date of admission	11-Feb 2020	18-Feb 2020	20-Feb 2020	25-Feb 2020	26-Feb 2020	10-Mar 2020	16-Mar 2020
Age (years)	60	71	67	78	54	49	61
Gender	Female	Male	Male	Male	Male	Female	Male
Cancer type	Breast cancer	Lung cancer	Oropharyngeal cancer	Lung cancer	Liver cancer	Colorectal cancer	Esophageal cancer
Smoking status	No	Yes	Yes	No	Yes	No	Yes
Epidemiological history	No	No	No	No	No	No	No
Other diseases	Hypertension	Liver cancer	Parotid gland carcinoma	Colorectal cancer and liver cancer	No	Hypertension	Hypertension
Treatment History	Surgery 2 weeks ago	Chemotherapy 1 week ago	Chemotherapy and immunotherapy 1 week ago	Newly diagnosed	Newly diagnosed	Newly diagnosed	Neoadjuvant chemotherapy 1 month ago
Symptoms							
Fever	No	No	No	Yes(38.3 °C)	Yes(39.7 °C)	No	No
Cough	No	Yes	Yes	Yes	No	No	Yes
Fatigue or myalgia	No	Yes	No	No	No	No	Yes
Expectoration	No	Yes	No	Yes	No	No	No
Dyspnea	No	Yes	No	No	No	No	No
Headache	No	No	No	No	No	No	Yes
Diarrhea	No	No	No	No	No	No	No
White blood cell count (× 10^9^ cells per L)	7.3	5.4	5.8	5.2	6.8	3.8	4.6
Low leukocyte count (< 9.5 × 10^9^ cells per L)	No	No	No	No	No	No	No
Lymphocyte count (× 10^9^ cells per L)	1.6	1.2	1.1	1.1	1.9	1.8	0.9
Lymphopenia (< 10^9^ cells per L)	No	No	No	No	No	No	Yes
CRP (mg/L)	1.84	2.45	2.2	7.73	9.39	1.07	0.49
Elevated CRP (> 10 mg/L)	No	No	No	No	No	No	No
LDH (U/L)	226	289	256	139	188	160	184
Elevated LDH (> 240 U/L)	No	Yes	Yes	No	No	No	No
ALT (U/L)	63	17	58	8	15	15	12
Elevated ALT (> 50 U/L)	Yes	No	Yes	No	No	No	No
AST (U/L)	38	22	54	14	47	22	16
Elevated AST (> 40 U/L)	No	No	Yes	No	Yes	No	No
1st round of PCR							
RNA-dependent RNA polymerase (RdRp)	Negative	Negative	Negative	Negative	Negative	Negative	Negative
Nucleocapsid (N) gene	Positive	Positive	Positive	Positive	Positive	Positive	Positive
2nd round of PCR							
RNA-dependent RNA polymerase (RdRp)	Negative	Negative	Negative	Negative	Negative	Negative	Negative
Nucleocapsid (N) gene	Positive	Positive	Positive	Negative	Negative	Positive	Positive
**CT evidence of pneumonia**							
Typical signs of viral infection	Positive	Negative	Negative	Negative	Negative	Negative	Negative
**Follow-up Treatment**							
	Transfer to a designated hospital	Chemotherapy	Transfer to a local hospital	Anti-infection therapy	Anti-infection therapy and chemotherapy	Laparoscopic radical resection	Chemotherapy

One laboratory-confirmed case of COVID-19 was found among 4113 patients (Table 5). This 61-year-old female outpatient with recurrent ovarian cancer lived in Jiaxing, Zhejiang province and received chemotherapy 2 weeks ago. The patient had a normal clinical examination and showed no clinical symptoms of COVID-19. She was quarantined at home and monitored by the local community after received the testing result. After 4 weeks of follow-up, the patient, her family, and individuals with intimate contact showed no clinical symptoms of COVID-19. The patient received a negative nucleic acid testing result of COVID-19 and normal chest CT image in our hospital before she got the next round of chemotherapy. This patient was diagnosed as a cancer patient with asymptomatic COVID-19 infection according to the COVID-19 Prevention and Control Guideline from CDC, China, 6th Edition (http://www.chinacdc.cn/jkzt/crb/zl/szkb_11803/jszl_11815/202003/W020200309376009304000.pdf).

**Table 5:** Clinical and laboratory characteristics of the case

Clinical characteristics	Values
Residence	Jiaxing
Date of admission	5-Mar 2020
Age (years)	61
Gender	Female
Cancer type	Ovarian cancer (relapse)
Smoking status	No
Epidemiological history	No
Complications	No
Cancer treatment regimen	Chemotherapy 2 weeks ago
Symptoms	
Fever	No
Cough	No
Fatigue or myalgia	No
Expectoration	No
Dyspnea	No
Headache	No
Diarrhea	No
**Laboratory characteristics**	
White blood cell count (× 10^9^ cells per L)	8.0
Low leukocyte count (< 9.5 × 10^9^ cells per L)	No
Lymphocyte count (× 10^9^ cells per L)	1.2
Lymphopenia (< 10^9^ cells per L)	No
CRP (mg/L)	0.19
Elevated CRP (> 10 mg/L)	No
LDH (U/L)	191
Elevated LDH (> 240 U/L)	No
ALT (U/L)	10
Elevated ALT (> 50 U/L)	No
AST (U/L)	16
Elevated AST (> 40 U/L)	No
Confirmatory test done (SARS-CoV-2 RT-PCR)	
RNA-dependent RNA polymerase (RdRp)	Positive
Nucleocapsid (N) gene	Positive
**CT evidence of pneumonia**	
Typical signs of viral infection	Negative

*CRP* C-reactive protein. *LDH* Lactate dehydrogenase. *ALT* Alanine transaminase. *AST* Aspartate transaminase

## Discussion

During the study period, despite the enormous challenges brought by the COVID-19 epidemic, our hospital still provided timely oncology or medical services to a large number of cancer patients. No COVID-19 case was found in the 3697 inpatients, medical staff or visitors. The practice has proved that our COVID-19 infection intervention measures, combined with the extensive SARS-CoV-2 nucleic acid testing in Zhejiang Provincial Cancer Hospital, not only effectively prevented and contained the spread of the virus, but also greatly promoted patient care. Our screening strategy relied on the adequate laboratory testing capacity, which brought high work load to the clinical laboratory staff. Other cancer centers suggested to test cancer patients with active therapy only [21], which could reduce the sample volume for the clinical laboratory, but we suggested additional prevention method must be strictly used to prevent in-hospital transmission by outpatients and patients’ companions, especially in region with a high incidence of SARS-CoV-2 infection.

There are some similar clinical features between patients with COVID-19 and cancer patients, including fever and low leukocyte count caused by cancer or chemotherapy. With the help of chest CT images, oncologists assess the course of fever and the pulmonary infection [7, 8, 22]. Nevertheless, it is challenging to differentiate COVID-19 pneumonia from aspiration pneumonia and radiation-induced pneumonitis. In this study, nucleic acid tests of SARS-CoV-2 were regarded as the gold standard of COVID-19 case diagnosis. Patients with positive chest CT with other clinical evidences were diagnosed as clinically suspected COVID-19 cases. Only 4 clinically suspected COVID-19 cases were found from February 1 to April 3 in Zhejiang Cancer Hospital during the outbreak of COVID-19. Three of them were tested negatively by the PCR method, and after at least 2 weeks of monitoring by local communities or hospitals, none of them has be diagnosed as infected patients.

Furthermore, for the other 4101 RNA negative patients, most of them were tested and excluded 2 weeks ago in our hospital. As we knew by now, none of them was diagnosed as COVID-19 cases by local hospitals and reported to the system. However, the PCR method still has a chance to produce false-negative results due to many reasons. A study presented five patients with confirmed COVID-19 infection and negative RT-PCR testing, which indicated that insufficient viral samples and laboratory difficulties might be responsible for the false-negative rate of SARS-CoV-2 RT-PCR testing [23]. Previously published research suggested that sputum showed a higher positive rate than pharyngeal swabs [24]. We changed our sample type from pharyngeal swabs to sputum during our study because the sputum samples have reported advantages and can be harvested by patients themselves, which reduced medical staff’s workload and exposure risks. For some patients who have no sputum, saliva from throat could still be used for testing because live virus can be found in saliva samples [25].

Cancer patients are known to be particularly susceptible to pneumonia because of weakened immune reaction to virus. For example, the 2009 H1N1 influenza pandemic caused a 31% ICU admission rate and a 22% mortality rate among patients with hematologic malignancies and/or hematopoietic stem cell transplant [26]. These outcomes are worse than the 25% ICU admission rate and a 7% mortality rate reported for healthy individuals infected with 2009 H1N1 influenza [27]. A study in *The Lancet Oncology* showed that cancer patients had a higher risk of COVID-19 and worse prognosis than patients without cancer [16]. Of 1590 cases with confirmed COVID-19, 18 patients had a history of cancer. Due to cancer heterogeneity and different treatment regimens, the conclusion from such a small sample size could not be generalized to all cancer patients [17, 18]. Furthermore, only patients with severe symptom were hospitalized during the early Wuhan pandemic due to the limited medical resources. In this cohort, the 18 cancer patients were significantly older than others, which could be a reason why they were hospitalized due to age-related symptoms. A letter in *The JAMA Oncology* pointed out that compared with individuals in the community, cancer patients at a tertiary hospital in Wuhan had an increased risk of COVID-19. Due to the high prevalence of COVID-19 in Wuhan, 41.3% of infected patients in this cohort might be related to hospital-acquired transmission. The fact that cancer patients visited the hospital more frequently than others could cause their infection [19]. In addition to the nucleic acid test of SARS-CoV-2, Chest CT images, which were considered highly accurate and efficient, were also recommended to be used as the diagnostic criteria for COVID-19 in the highly epidemic area of Wuhan. Among these 12 patients, eight patients confirmed by chest CT showed negative RT-PCR results [19]. We conducted extensive COVID-19 screening of 3919 cancer patients mainly from Zhejiang province in China and found one asymptomatic patient. Interestingly, in Zhejiang province, five cancer patients with typical CT images of viral infection were finally excluded, which showed that CT images could not be used as the primary diagnostic basis in moderate epidemic areas. On the other hand, by April 17, the total number of local COVID-19 cases reported by this province with a population of 50.00 million (2018) was 1182, with another 50 imported cases and 36 cases from an outbreak in a jail. It can be reasonably inferred that cancer patients may not be more prone to COVID-19 than the general population in this area.

A viewpoint in *The JAMA Oncology* recommended several mandatory measures in a National Cancer Center of China [15]. A total of 2795 outpatients received clinic consultation, chemotherapy, immunotherapy, and radiotherapy. Only 149 cancer patients who need emergency surgeries were admitted into the wards. These infection-control interventions reduced non-emergency surgery and oncology service, which may worsen the prognosis of many cancer patients. Moreover, the asymptomatic and subclinical infection may be missed because the nucleic acid test of SARS-CoV-2 was only assessed among few patients with clinically suspected COVID-19. Interestingly, our findings indicated that 5 cancer patients without clinically suspected COVID-19 were confirmed positive N genes, and another 1 patient was laboratory confirmed COVID-19 with asymptomatic symptom. Therefore, we suggested that the nucleic acid of SARS-CoV-2 should be assessed among all cancer patients due to the asymptomatic and subclinical infection.

The treatment of cancer patients diagnosed with COVID-19 is complicated. Local guidelines for COVID-19 prevention and control recommended that all patients, cancer patients included, with confirmed infections, must be transferred to designated hospitals with multidisciplinary medical teams. According to the tumor’s biological characteristics, the clinical condition of the patients, the treatment characteristics (i.e., expected benefits and adverse events such as immunosuppression), and the response to current anti-cancer treatments, the infected cancer patients were treated on a personalized basis. First, timely anti-infection and supportive treatment was provided. Chemotherapy and elective surgery that led to immunosuppression were delayed until the patient recovered from SARS-CoV-2 infection, which was defined by symptom relief and two consecutive negative RT-PCR tests. During anti-infective treatment, cancer-related life-threatening symptoms, such as airway obstruction, internal bleeding must be monitored and treated. Other cancer centers have adopted similar strategies to treat infected cancer patients [28–30].

One caveat of our study is that the majority of patients enrolled had solid tumors. Therefore, malignancies with severe immunosuppression, such as hematologic malignancies and pediatric tumors, have not been evaluated. Also, most patients came from Zhejiang province, which was an intermediate epidemic region, with the prevalence much lower than epicenters like Wuhan.

## Conclusions

Thanks to the tremendous and active efforts made to treat infected COVID-19 patients and to quarantine suspected patients and their contacts, new cases of COVID-19 are rarely diagnosed daily in China recently. Our study suggested that a comprehensive set of virus preventive interventions combined with SARS-CoV-2 nucleic acid test could effectively triage and manage cancer patients, and ultimately promote patient care. Cancer patients in Zhejiang were not at a higher risk to COVID-19 than the general population.

## Supplementary Information

**Additional file 1 Supplementary Table S1**. Graded protection requirements for medical staff.

## Data Availability

The raw data is available by contacting corresponding author.

## Abbreviations

COVID-19: Coronavirus disease 2019
SARS-CoV-2: Severe acute respiratory syndrome coronavirus 2
CDC: Center of disease control
RdRp: RNA-dependent RNA polymerase
N: Nucleocapsid
E: Envelope
CRP: C-reactive protein
LDH: Lactate dehydrogenase
ALT: Alanine aminotransferase
AST: Aspartate aminotransferase
