# Supplementary material for: Cancer patient management strategy in a Cancer Center of Zhejiang, China during the COVID-19 pandemic
Source: BMC Cancer. 2020 Dec 7;20:1194. doi: 10.1186/s12885-020-07577-8 (PMC7719731; doi:10.1186/s12885-020-07577-8)
Supplement: Supplementary file 1 — Additional file 1 Supplementary Table S1. Graded protection requirements for medical staff. [file 12885_2020_7577_MOESM1_ESM.docx]

Supplementary table1: Graded protection requirements for medical staff

| Protection levels | | Level 1 | | | Level 2 | | | Level 3 |
| --- | --- | --- | --- | --- | --- | --- | --- | --- |
| Service conditions | | General outpatient clinics | General wards | Preview and triage | Fever clinics | Isolation wards | Provide general medical services | Aerosol-generating medical procedures |
| Patients | | Routine patients | | All patients | Patients had a fever | Suspected and confirmed COVID-19 | | Suspected and confirmed COVID-19 |
| Protective equipment | Hand hygiene | **+** | | **+** | **+** | | | **+** |
|  | Hospital cap | ± | | **+** | **+** | | | **+** |
|  | Surgical mask | **+** | | **+** | - | | | - |
|  | Respirator N95 | - | | - | **+** | | | **+** |
|  | Face shields or goggles | - | | - | ± | | | **+** |
|  | Hospital uniform | **+** | | **+** | **+** | | | **+** |
|  | Isolation gown | - | | **+** | * | | | - |
|  | Protective clothing | - | | - | * | | | **+** |
|  | Latex gloves | **±** | | **+** | **+** | | | **+** |
|  | Shoe cover | - | | - | **+** | | | **+** |

Note: "+" should wear protective equipment; "-" do not need to wear protective equipment; "±" should wear when contact patients closely; "*" wear either impermeable isolation gown or protective clothing.
